# D-mannose-Coating of Maghemite Nanoparticles Improved Labeling of Neural Stem Cells and Allowed Their Visualization by *ex vivo* MRI after Transplantation in the Mouse Brain

**DOI:** 10.1177/0963689719834304

**Published:** 2019-07-11

**Authors:** Igor M. Pongrac, Marina Dobrivojević Radmilović, Lada Brkić Ahmed, Hrvoje Mlinarić, Jan Regul, Siniša Škokić, Michal Babič, Daniel Horák, Mathias Hoehn, Srećko Gajović

**Affiliations:** 1 University of Zagreb School of Medicine, Croatian Institute for Brain Research, Zagreb, Croatia; 2 Institute of Macromolecular Chemistry, Academy of Sciences, Prague, Czech Republic; 3 Max Planck Institute for Metabolism Research, In-vivo-NMR Laboratory, Cologne, Germany

**Keywords:** neural stem cells, magnetic resonance imaging, brain, mouse, nanoparticles, maghemite

## Abstract

Magnetic resonance imaging (MRI) of superparamagnetic iron oxide-labeled cells can be used as a non-invasive technique to track stem cells after transplantation. The aim of this study was to (1) evaluate labeling efficiency of D-mannose-coated maghemite nanoparticles (D-mannose(γ-Fe_2_O_3_)) in neural stem cells (NSCs) in comparison to the uncoated nanoparticles, (2) assess nanoparticle utilization as MRI contrast agent to visualize NSCs transplanted into the mouse brain, and (3) test nanoparticle biocompatibility. D-mannose(γ-Fe_2_O_3_) labeled the NSCs better than the uncoated nanoparticles. The labeled cells were visualized by *ex vivo* MRI and their localization subsequently confirmed on histological sections. Although the progenitor properties and differentiation of the NSCs were not affected by labeling, subtle effects on stem cells could be detected depending on dose increase, including changes in cell proliferation, viability, and neurosphere diameter. D-mannose coating of maghemite nanoparticles improved NSC labeling and allowed for NSC tracking by *ex vivo* MRI in the mouse brain, but further analysis of the eventual side effects might be necessary before translation to the clinic.

## Introduction

Stem cell therapies are a promising area of regenerative medicine being already tested in multiple clinical trials. In particular for neurological diseases, stem cells offer the potential to contribute to brain repair or even replace the lost neurons. Recent studies show that neural stem cells (NSCs) can enhance functional recovery after stroke via secretion of neurotrophic factors, immunomodulation, and stimulation of endogenous neurogenesis and neovascularization^1–4^. Similar therapeutic strategies could be applied in the treatment of spinal cord injury, retinal degenerative disease, Alzheimer’s disease, Parkinson’s disease, and amyotrophic lateral sclerosis or other neurodegenerative diseases^5–8^.

An essential point to understand better the mechanisms of action along with benefits of stem cell therapies would be the ability to monitor longitudinally the spatiotemporal dynamics of these cells *in vivo*, ideally through non-invasive imaging systems. Magnetic resonance imaging (MRI), as a standard clinical tool in neurological assessment, is particularly suitable for monitoring cell distribution and engraftment during the early phase after transplantation^9–16^. MRI allows visualization of hydrogen atom distribution in tissues which differ in water composition depending on their unique macromolecular structure. To enable more sensitive and specific diagnostic information, MRI-specific contrast agents can be used to alter the tissue proton relaxivity modifying the surrounding MR signal. Contrast agents can be used for cell-tracking purposes if applied as cellular labels prior to transplantation. However, there is a gradual decrease in hypointensity over time, which could be indicative of remaining cell locations but still lack information about cell viability or functional state. Early studies used gadolinium rhodamine dextran-based contrast agents to monitor cell migration *in vivo.* However, deleterious effects were shown after long-term monitoring of transplanted gadolinium rhodamine dextran-labeled cells in a rat model of stroke which resulted in a slight increase in lesion size compared with non-treated stroke-only animals^17^. Stem cell therapeutic potential depends on their full capabilities to migrate to the site of injury, integrate, differentiate at the part of the tissue of interest, and produce and release bioactive molecules. Subsequently, any alterations of this potential by cell-labeling strategies must be carefully evaluated^18^.

Different superparamagnetic iron oxide nanoparticles (SPIONs) such as Endorem and Sinerem from Guerbet, or Resovist and Supravist from Bayer, have been tested in clinical trials, but all were discontinued due to financial reasons^19,20^. SPIONs shorten T_2_ relaxation time, allowing their hypointense signal detection inside the tissue^21–23^. There are some limitations in labeling stem cells with magnetic contrast agents. The gradual loss of hypointense signal could be due to fast cell proliferation after transplantation, or loss of iron oxide due to cell death and SPION internalization by endogenous microglia or macrophages^15^. False positive MRI results could occur due to possible micro-bleeding and ferritin deposition at the injury site, or due to iron oxide distribution in the extracellular space^15,16,24^. Despite the abovementioned limitations in labeling stem cells with magnetic contrast agents, there are still unquestionable strengths of short-term MR-imaging and real-time MR-guided delivery of cellular therapeutics. For example, it has been shown that high-speed real-time MRI can be used to visualize the intravascular distribution of a superparamagnetic iron oxide contrast agent that could accurately predict the distribution of intra-arterial administered stem cells to the brain^25,26^. Another advantage would be the usage of a new magnetic particle imaging (MPI) technology, which allows direct and quantitative imaging of SPION-labeled cell distribution^27–29^. In ideal applications, SPIONs would have a narrow size distribution, be monodispersed, homogeneously composed, and coated with materials which make them stable, biocompatible, and biodegradable^23,30^. In order to design nanoparticles with reduced toxicity and improved labeling efficacy, a detailed characterization of a material’s biocompatibility is of critical importance. Moreover, cell type-specific nanosafety optimization studies are needed due to demonstrated cell type-associated diversity in nanoparticle-evoked responses^31–34^.

In the present study, maghemite (γ-Fe_2_O_3_) nanoparticles coated with D-mannose (D-mannose(γ-Fe_2_O_3_)) were tested as a candidate for neural stem cell labeling and tracking by MRI. D-mannose is a common sugar existing in various foods, which plays an important role in the immune system as a component of the innate immune system mannose-binding lectin (MBL)^35–39^. D-mannose is widely used as an inexpensive backbone for the synthesis of immunostimulatory and antitumor agents, in novel non-viral gene therapy approaches, and as a mediator in natural killer cell function^39–44^. D-mannose is a promising candidate for nanoparticle surface coating^45^. D-mannose-modified iron oxide nanoparticles are internalized by rat bone marrow stromal cells or synaptosomes, which can be further manipulated by an external magnetic field^46^.

In the present study, our aim was to verify whether D-mannose coating of maghemite nanoparticles (D-mannose(γ-Fe_2_O_3_)) improved labeling of mouse NSCs to be visualized by MRI and to evaluate their biocompatibility in comparison to the uncoated counterparts.

## Materials and Methods

### Synthesis and Characterization of Nanoparticles

The D-mannose-modified/coated maghemite nanoparticles (D-mannose(γ-Fe_2_O_3_)) and unmodified/uncoated maghemite nanoparticles (Uncoated(γ-Fe_2_O_3_)) were prepared by *in situ* precipitation of iron oxide in D-mannose solution method as described previously^47^. Briefly, γ-Fe_2_O_3_ nanoparticles were obtained by chemical co-precipitation of FeCl_2_ and FeCl_3_, followed by oxidation of the produced magnetite with sodium hypochlorite to maghemite (γ-Fe_2_O_3_). γ-Fe_2_O_3_ nanoparticles were coated post-synthesis with D-mannose^45^. Detailed examination and characterization of the nanoparticles after synthesis was done by transmission electron microscopy (TEM) as described previously^45,48,49^. Briefly, the morphology of the particles was evaluated at 120 kV using a Tecnai Spirit G2 transmission electron microscope (FEI, Brno, Czech Republic) and the micrographs processed by NIS Elements image analysis program (Laboratory Imaging, Prague, Czech Republic).

### Animals

The mouse inbred strain C57Bl/6NCrl was used. The animals were housed in a temperature (22 ± 2°C) and humidity controlled environment, under 12/12 hours light/dark cycles. Water and pelleted food were given *ad libitum*. All animal procedures were approved by the internal review board of the ethics committee of the School of Medicine University of Zagreb and were in accordance with the ethical codex of the Croatian Society for Laboratory Animal Science and with EU Directive 2010/63/EU on the protection of animals used for scientific purposes.

### Neural Stem Cell Culture and Treatment

Neural stem cells were isolated from mouse fetuses at gestational day 14.5 (E14.5) as described previously^50–52^. Briefly, pregnant females were sacrificed and neural stem cells were isolated from the telencephalic wall of E14.5 fetuses by microdissection and dissociation using StemPro Accutase (Gibco by Thermo Fisher Scientific, Waltham, MA, USA). Individual neural stem cells were obtained by trituration. Cells were maintained at 37°C in a humidified atmosphere with 5% CO_2_/95% O_2_. Expansion medium contained: DMEM/F-12 with GlutaMAX (Gibco by Life Technologies), 1% N2 Supplement (Gibco by Life Technologies), 2% B27 supplement (Gibco by Life Technologies), 1% penicillin/streptomycin (Gibco by Life Technologies), recombinant mouse epidermal growth factor (EGF) 20 ng/ml (Gibco by Life Technologies) and recombinant mouse basic fibroblast growth factor (bFGF) 10 ng/ml (Gibco by Life Technologies). Cells were cultivated in suspension. After 2 days neurospheres were formed. Neurospheres were dissociated using StemPro Accutase (Gibco by Thermo Fisher Scientific, Waltham, MA, USA), plated on 6-well plates at 2×10^5^ NSC/well, and allowed to attach for 24 h for Prussian blue staining, TEM, and flow cytometry experiments. All plates were previously coated for 12 h with 50 µg/ml poly-D-lysine water solution (Sigma-Aldrich, Merck KGaA, Darmstadt, Germany). For intracerebral transplantation purpose, neurospheres were dissociated by StemPro Accutase and 200,000 cells were transplanted in 1 μl of cell medium. Neurospheres were dissociated with StemPro Accutase and plated on 24-well plates at a cell density of 4×10^4^ NSC/well for MTT (3-[4,5-dimethylthiazol-2-yl]-2,5- diphenyl tetrazolium bromide) cell viability assay. Cells were cultivated as free-floating aggregates in suspension for neurosphere assay purpose. Neurospheres were dissociated, single cells and were plated on uncoated 6-well plates (250,000 cells per well), and were allowed to develop into neurospheres in a humidified atmosphere with 5% CO_2_ at 37°C.

Neurospheres were first dissociated using StemPro Accutase, single cells and small neurospheres were plated on 12 mm coverslips (250,000 cells per coverslip) previously coated with Poly-d-lysine (500 μg/ml, 24 h at 37°C, Sigma-Aldrich) and laminin (10 μg/ml, 24 h at 37°C, Sigma-Aldrich) for the purpose of differentiation analyses. To the cells used for *in vitro* proliferation experiments, Uncoated(γ-Fe_2_O_3_) or D-mannose(γ-Fe_2_O_3_) nanoparticles were added for 48 h and left to proliferate for an additional 48 h in a medium with proliferation factors. Cells were fixed with 4% paraformaldehyde (PFA) (pH 7.4) on the 5th day after plating. For *in vitro* differentiation experiments Uncoated(γ-Fe_2_O_3_) or D-mannose(γ-Fe_2_O_3_) nanoparticles were added for 48 h to cells left to proliferate for an additional 5 days in a medium without proliferation factors. Cells were fixed with 4% PFA (pH 7.4) on the 8th day after plating.

D-mannose(γ-Fe_2_O_3_) and Uncoated(γ-Fe_2_O_3_) nanoparticles were added directly to the culture medium 24 h after NSC plating and incubated for 48 h. D-mannose(γ-Fe_2_O_3_) and Uncoated(γ-Fe_2_O_3_) nanoparticles were used in the following concentrations: 0.002, 0.01, 0.02, 0.03, 0.04, 0.1, 0.15, and 0.2 mg/ml. The nanoparticles were not added to the control unlabeled cells.

To determine the mechanism of nanoparticle uptake, after seeding and attaching NSCs were pre-treated with various inhibitors of endocytosis for 30 min and then incubated with 0.2 mg/ml of D-mannose(γ-Fe_2_O_3_) or Uncoated(γ-Fe_2_O_3_) nanoparticles for the next 48 h in the presence of the inhibitor^53^. The inhibitors used were: phenyl arsine oxide (12 nM, Sigma-Aldrich), cytochalasin D (60 nM, Sigma-Aldrich), nocodazole (20 nM, Sigma-Aldrich), and filipin (0.3 µg/ml, Sigma-Aldrich).

### Prussian Blue Staining

After a 48 h incubation period the medium with nanoparticles was removed, cells were washed three times with phosphate-buffered saline (PBS), fixed with 4% PFA (Sigma-Aldrich) for 20 min and stained with 1:1 mixture of 10% K_4_Fe(CN)_6_ (Sigma-Aldrich) and 20% HCl for 20 min. Cells were counterstained with 0.1% Nuclear Fast Red (Sigma-Aldrich) for 1 min, mounted with HistoMount (Invitrogen by Thermo Fisher Scientific, Waltham, MA, USA) and coverslipped. After drying, the cells were analyzed under bright field using an ECLIPSE E200 light microscope (Nikon Instruments, Tokyo, Japan).

### Flow Cytometry

After labeling, the cells were dissociated with StemPro Accutase (Life Technologies), washed once with PBS, resuspended in PBS containing 2% FBS and 2 mM EDTA (pH 7.4) and passed through a 40 µm *Falcon™ cell strainer* (Fisher Scientific by Thermo Fisher Scientific, Waltham, MA, USA). To determine the percentage of cells labeled with nanoparticles, the increase of the side scattered (SSC) light of the laser beam was measured using the Attune® Acoustic Focusing Flow Cytometer (Applied Biosystems, Foster City, CA, USA). The intensity of the SSC is proportional to the intracellular density^54^. The percentage of positive cells was determined with FCS Express 4 software (De Novo Software, Glendale, CA, USA) using the Overton cumulative histogram subtraction method^55^.

The effects of inhibitors on cellular uptake of nanoparticles were examined using Attune® Acoustic Focusing Flow Cytometer and FlowJo vX.0.7 software (Tree Star, Inc., Ashland, OR, USA).

### Transmission Electron Microscopy

The cells treated by nanoparticles were detached from the surface by cell dissociation reagent StemPro Accutase (Life Technologies), washed once with DMEM/F-12 medium, shortly centrifuged and fixed overnight with 2% glutaraldehyde. The fixed cells were washed 3 times 15 min each with 0.1 M phosphate buffer (PB), post-fixed in 1% osmium tetroxide in 0.1 M PB for 1 h, washed 3 times 15 min each with 0.1 M PB and rinsed with water for 10 min. After rinsing, NSCs were immersed in 2% uranyl acetate in water for 1 h, then dehydrated in graded series of ethanol (20%, 50%, 70%, 90%, 15 min each), followed by two 100% ethanol washes, and two 15 min acetone washes. After each step the cells were centrifuged for 1 min at 1,500 *g* to settle, the supernatant removed, and the solution changed. For embedding in the Durcopan (Merck KGaA, Darmstadt, Germany) the cells were placed in 1:1 mixture of acetone/Durcupan resin for 3 h at room temperature, after which they were transferred to 100% Durcupan resin, for 72 h polymerization at 64°C.

Using a diamond knife (DiATOME) on an ultramicrotome RMC Power Tome XL (Boeckeler Instruments, Tucson, AZ, USA) semi-thin sections were cut and stained with 0.2% toluidine blue solution (Sigma-Aldrich). Sections were examined under a light microscope (ECLIPSE E200, Nikon Instruments). Subsequently, from selected samples 70 nm ultra-thin sections were cut, picked up on copper grids, and contrasted with 2% uranyl acetate (Merck) and Reynolds lead citrate. The sections were examined using a TEM902A transmission electron microscope (Zeiss, Oberkochen, Germany) operated at 80 kV, using magnifications ranging from 7,000 to 30,000.

### Stereotaxic Transplantation of Neural Stem Cells Into the Mouse Brain

Together with nanoparticle labeling, the cells were treated just prior to transplantation with PKH26 fluorescent dye (PKH26 Red Fluorescent Cell Linker Kit for general cell membrane labeling, Sigma-Aldrich) following the manufacturer’s instructions. NSC were dispersed and resuspended in Hank's balanced salt solution (HBSS, Invitrogen). Animals were anesthetized with an intraperitoneal injection of Avertin (Sigma-Aldrich) at a dose of 0.5 g/kg and fixed in a stereotactic frame (KOPF stereotaxic apparatus 900LS). After exposing the skull by a small incision, a hole was drilled at the following coordinates (in mm) relative to bregma: anteroposterior −1.3, mediolateral +2.0 and dorsoventral −1.5 (from dura), determined according to the stereotaxic atlas^56^. We injected 2 μl of homogeneous cell suspension in HBSS buffer containing 400,000 of cells into the brain striatum through a Hamilton syringe needle, which was kept in place for 5 min before being slowly retracted. The wound was closed with silk suture and the animals were kept for an hour on a heating pad to recover prior to returning to their cages.

Mice were anesthetized using Avertin (0.5 g/kg) 72 hours after NSC transplantation and subsequently transcardially perfused with freshly prepared PB (0.1 M, pH 7.4) followed by buffered 4% PFA (in 0.1 M PB, pH 7.4). Brains were carefully dissected and post-fixed by immersion in the same fixative at 4°C overnight.

### Magnetic Resonance Imaging

To validate the MRI visibility of the analyzed D-mannose(γ-Fe_2_O_3_) nanoparticles in *ex vivo* mouse brain, the isolated brains were washed three times in PBS and transferred in 5 ml syringes filled with Fomblin (Solvay, Brussels, Belgium). A custom-made holder for the syringe was placed on a mouse holder (Medres, Cologne, Germany) and used in combination with a 9 cm resonator for transmission (Bruker, Ettlingen, Germany) and mouse quadrature surface coil (Bruker) for signal detection. All MR experiments were carried out on a Biospec 9.4 T animal scanner system with a 20 cm diameter bore magnet (Bruker) operated with ParaVision 5.1 software (Bruker). Transplanted cells were visualized performing a multi-slice multi-echo sequence using the following parameters: TR = 4,000 ms, TE = 12 ms, slice thickness = 0.7 mm, number of slices (coronal) = 10, FOV = 12×12 mm^2^, matrix = 160×160, resolution 0.075×0.075 mm^2^, bandwidth 50 kHz, echoes = 8. The acquisition time for these experiments was 10 min and 40 s. Quantitative T2 maps were calculated using a custom-made program developed in IDL (ITT Visual Information Solutions, Exelis Visual Information Soution, Boulder, CO, USA). The images obtained were analyzed with the ImageJ program (NIH, Bethesda, MD, USA).

### Immunohistochemistry and Prussian Blue Staining

After MRI the brains were washed in PBS and transferred to 30% sucrose in PBS at 4°C until sunk. Coronal 20 μm-thick sections were serially cut with a cryostat, mounted on Superfrost Plus slides (Menzel Glaser, Fisher Scientific, Loughborough, England), and used for immunohistochemistry and Prussian blue staining.

For Prussian blue staining, the selected sections were stained with a 1:1 mixture of 10% K_4_Fe(CN_)6_ (Sigma-Aldrich) and 20% HCl for 20 min. The sections were counterstained with 0.1% Nuclear Fast Red (Sigma-Aldrich) for 5 min, washed in PBS and distilled H_2_O, mounted with HistoMount (Invitrogen) and coverslipped. After air drying, the brain sections were analyzed under bright field using an ECLIPSE E200 light microscope (Nikon Instruments).

For immunohistochemistry and immunocytochemistry, polyclonal antibodies against nestin (mouse monoclonal, diluted 1:200, Millipore, MAB353), MAP2 (chicken polyclonal, diluted 1:1,000, Abcam plc., Cambridge, UK, ab5392), GFAP (chicken polyclonal ab, diluted 1:250, Abcam, ab4674) and for oligodendrocytes O4 (monoclonal mouse anti-O4, dilution 1:50; Merck Millipore KGaA, Darmstadt, Germany, MAB345) were used. Briefly, brain sections/cells were incubated overnight at room temperature with the primary antibodies diluted in 0.2% Triton X-100 (Sigma-Aldrich) in PBS and 1% specific serum. The next day the sections/cells on glass slides were rinsed three times with PBS and then incubated for 2 h with the secondary antibodies diluted 1:500 in 0.2% Triton X-100 in PBS (goat anti-mouse Alexa Fluor 488 (Invitrogen), goat anti-mouse Alexa Fluor 546 (Invitrogen) and goat anti-chicken Alexa Fluor 546 (Invitrogen)). Secondary antibodies were rinsed with PBS three times and 4′,6-diamidine-2′-phenylindole dihydrochloride (DAPI 250 ng/ml; Roche, Basel, Switzerland) was used as a nuclear counterstain. Finally, the brain sections/cells were rinsed in PBS, air dried, mounted with Dako Fluorescent Mounting and coverslipped before examination with the confocal microscope (Leica SP8 X FLIM, Germany).

### Nanoparticle Biocompatibility *In Vitro* Tests

The labeled cells were tested by MTT assay, CalceinAM/PI cytotoxicity assay and neurosphere assay.

MTT (Sigma-Aldrich) was added to the cell culture medium at concentration 0.5 mg/ml and the cells incubated for 45 min at 37°C in 5% CO_2_/95% O_2_. The formed formazan crystals were dissolved in DMSO (Sigma-Aldrich), after which optical density was measured at 595 nm using a Microplate reader (680 XR, Bio-Rad Laboratories, Japan). MTT data were expressed as a percentage of the average values of the control cells according to the equation:

$$Cell\;viability\;\left(\%\right)\;=\;\left(A_{595sample}-\;A_{595blank}\right)/\left(A_{595control}-\;A_{595blank}\right)\;*\;100$$

For the CalceinAM/PI cytotoxicity assay the dissociated cells were incubated with 0.1 µM calcein AM (Invitrogen) and 5 ng/ml propidium iodide (Invitrogen). The percentage of calcein AM-positive NSC was analyzed using Attune acoustic focusing cytometer (Applied Biosystems) and calculated using FlowJo vX.0.7 software.

For neurosphere assay, cells were cultivated as free-floating aggregates in suspension for 24 h in triplicates; 0.02 or 0.2 mg/ml of D-mannose(γ-Fe_2_O_3_) nanoparticles were added to the medium for 48 h. After 2 days the average size of the neurospheres were measured in 10 visual fields per well in triplicates.

### Statistical Analysis

For each experimental group, data were evaluated separately for a minimum of three independent experiments. For the nanoparticle uptake mechanism flow cytometry, the data were based on quadruplicate of each individual experiment. Data from the different experimental groups were statistically compared using one-way ANOVA analysis with Tukey’s test or Dunnet’s test as post-ANOVA analysis (*p* < 0.05) provided in the GraphPad Prism software (GraphPad Software, Inc., San Diego, CA, USA). Grubbs’ test provided in the GraphPad Prism software (GraphPad Software, Inc.) was used to compare groups in flow cytometry experiments. Data were presented as mean values ± standard deviation (SD).

## Results

### D-mannose(γ-Fe_2_O_3_) Nanoparticles Label NSCs More Efficiently than Uncoated(γ-Fe_2_O_3_) Nanoparticles

**Figure 1.**
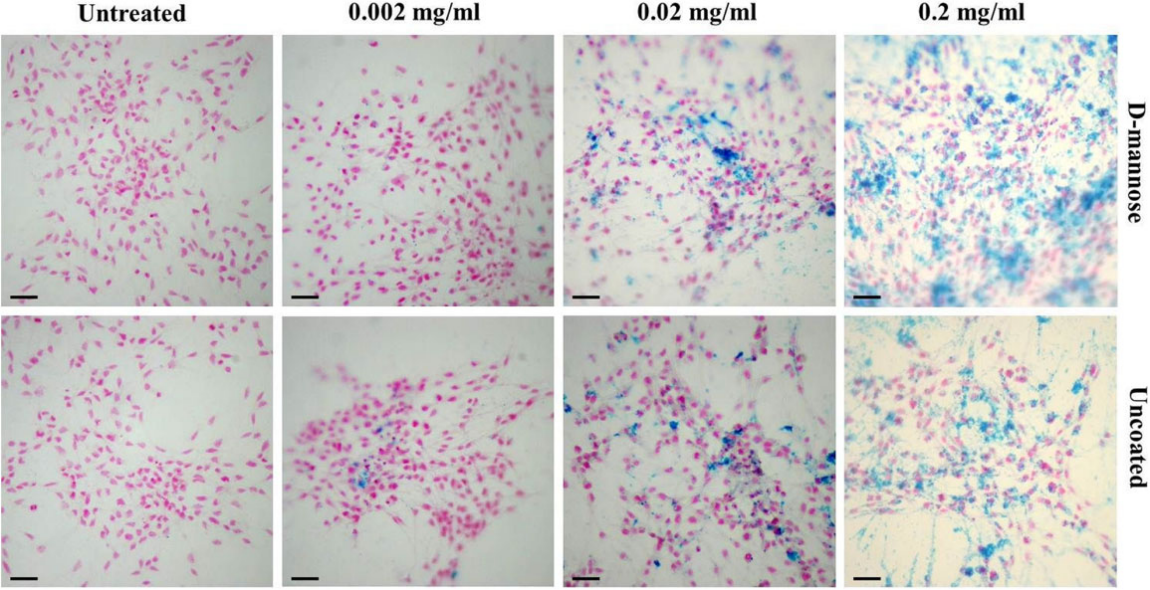
Nanoparticle internalization verified by Prussian blue staining. Prussian blue staining of neural stem cells labeled for 48 h with ascending concentrations of D-mannose(γ-Fe_2_O_3_) or Uncoated(γ-Fe_2_O_3_) nanoparticles. The blue precipitate represents the nanoparticles. Nuclear Fast Red staining showed the position of the nuclei. Scale bar: 50 µm.

To verify whether NSC labeling can be improved with D-mannose(γ-Fe_2_O_3_) in comparison to Uncoated(γ-Fe_2_O_3_) nanoparticles, the cells were treated for 48 h and subsequently stained by Prussian Blue (Fig. 1). The presence of nanoparticles within the NSCs was indicated by a formation of blue precipitates as a result of the reduction of ferric to ferrous iron. The results clearly indicate the presence of both nanoparticle types within the NSCs, but more abundant when labeled by the same concentration of D-mannose(γ-Fe_2_O_3_).

**Figure 2.**
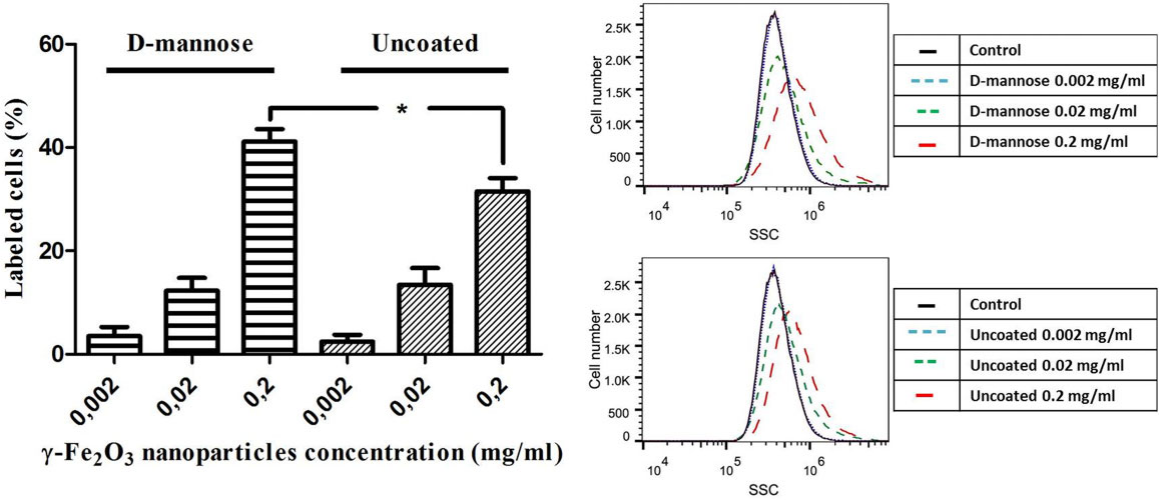
D-mannose(γ-Fe_2_O_3_) nanoparticles label neural stem cells (NSCs) more efficiently than Uncoated(γ-Fe_2_O_3_) nanoparticles. (A) Quantitative analysis of the changes in intracellular density of NSCs labeled with ascending concentrations of D-mannose(γ-Fe_2_O_3_) or Uncoated(γ-Fe_2_O_3_) nanoparticles for 48 h, performed by Overtone cumulative histogram subtraction of flow cytometry histograms. (B) Flow cytometry histograms of D-mannose(γ-Fe_2_O_3_) or Uncoated(γ-Fe_2_O_3_) nanoparticles labeling efficiency of NSCs (Black line – control, three blue lines – nanoparticle concentration of 0.002 mg/ml, two green lines – nanoparticle concentration of 0.02 mg/ml, one red line – nanoparticle concentration of 0.2 mg/ml). The asterisk indicates a statistically significant (*p* < 0.05) difference between same nanoparticle concentrations.

Flow cytometry measurements were performed to quantify the observed visual difference in NSC labeling. The changes of the laser beam SSC allowed measuring the changes in intracellular density, which correspond to nanoparticle internalization. The nanoparticle uptake was dose-dependent, and at concentrations of 0.2 mg/ml, D-mannose coating significantly improved nanoparticle internalization compared with their uncoated counterparts (Fig. 2).

**Figure 3.**
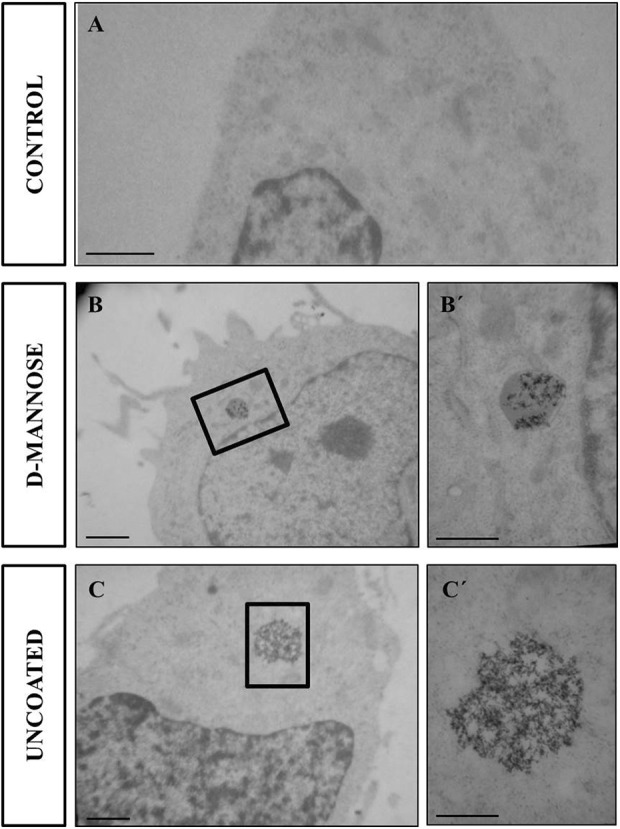
Transmission electron microscopy of neural stem cells (NSCs) nanoparticle internalization. Transmission electron micrographs of (A) unlabeled neural stem cells (NSCs), (B) labeled for 48 h with 0.02 mg/ml of D-mannose(γ-Fe_2_O_3_) nanoparticles or (C) Uncoated(γ-Fe_2_O_3_) nanoparticles. Inserts (B´, C´) show higher magnification of the nanoparticle aggregates inside the cell cytoplasm. Scale bar: 1 μm (A, B, C), 500 nm (B´, C´).

Transmission electron microscopy was used to confirm the internalization of D-mannose(γ-Fe_2_O_3_) or Uncoated(γ-Fe_2_O_3_) nanoparticles in the NSCs. TEM micrographs clearly displayed and confirmed that both D-mannose-coated and uncoated nanoparticles were located intracellularly (Fig. 3). After 48 h incubation with D-mannose(γ-Fe_2_O_3_) nanoparticles, nanoparticle aggregates were localized in structures surrounded by a membrane, probably trafficking toward lysosomes (Fig. 3B). In contrast to D-mannose(γ-Fe_2_O_3_) nanoparticles, Uncoated(γ-Fe_2_O_3_) nanoparticles were not found inside membrane-bound vesicles; instead, they were found as aggregates dispersed in the cell cytosol (Fig. 3C´). The nanoparticles were loosely arranged in groups, and individual black dots of particles could still be observed. We did not detect any nanoparticles adhered on top of the cell membrane.

**Figure 4.**
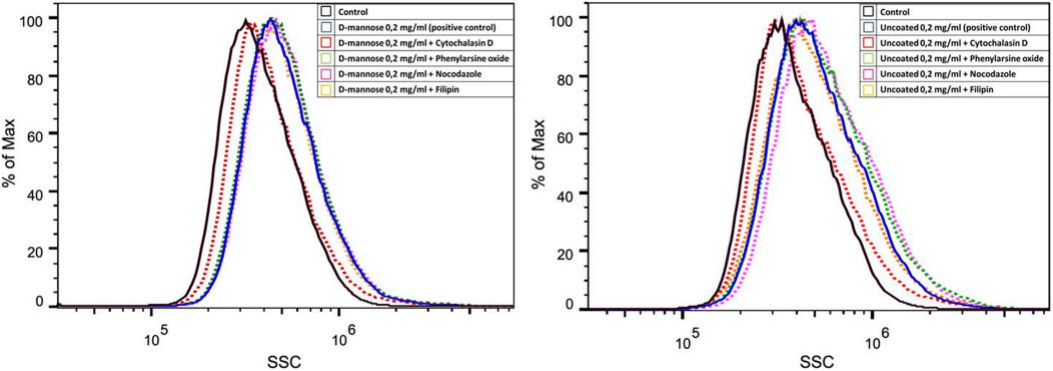
Neural stem cells (NSCs) internalize D-mannose(γ-Fe_2_O_3_) and Uncoated(γ-Fe_2_O_3_) nanoparticles through macropinocytosis. Flow cytometry histograms of NSCs labeled with D-mannose(γ-Fe_2_O_3_) or Uncoated(γ-Fe_2_O_3_) nanoparticles show changes in side scattered (SSC) light of the laser beam after pre-treatment with different endocytosis inhibitors: cytochalasin D (red), phenylarsine oxide (black), nocodazole (pink), and filipin (yellow). Unlabeled cells as controls (black), and non-pre-treated labeled cells as positive controls (blue).

To clarify which endocytotic pathway was involved in NSC internalization of D-mannose(γ-Fe_2_O_3_) and Uncoated(γ-Fe_2_O_3_) nanoparticles, different inhibitors of endocytosis were applied prior to nanoparticle treatment and their effects evaluated by flow cytometry. The NSCs treated with an inhibitor of actin-dependent macropinocytosis, cytochalasin D, decreased labeling, being unable to internalize the nanoparticles. No changes in labeling were found when phenylarsine oxide, nocodazole, or filipin were applied (Fig. 4). This indicated that the internalization of both types of nanoparticles was via actin-dependent macropinocytosis.

### D-mannose(γ-Fe_2_O_3_)-Labeled NSCs can be Efficiently Detected by *ex vivo* MRI after Transplantation Into the Mouse Brain

**Figure 5.**
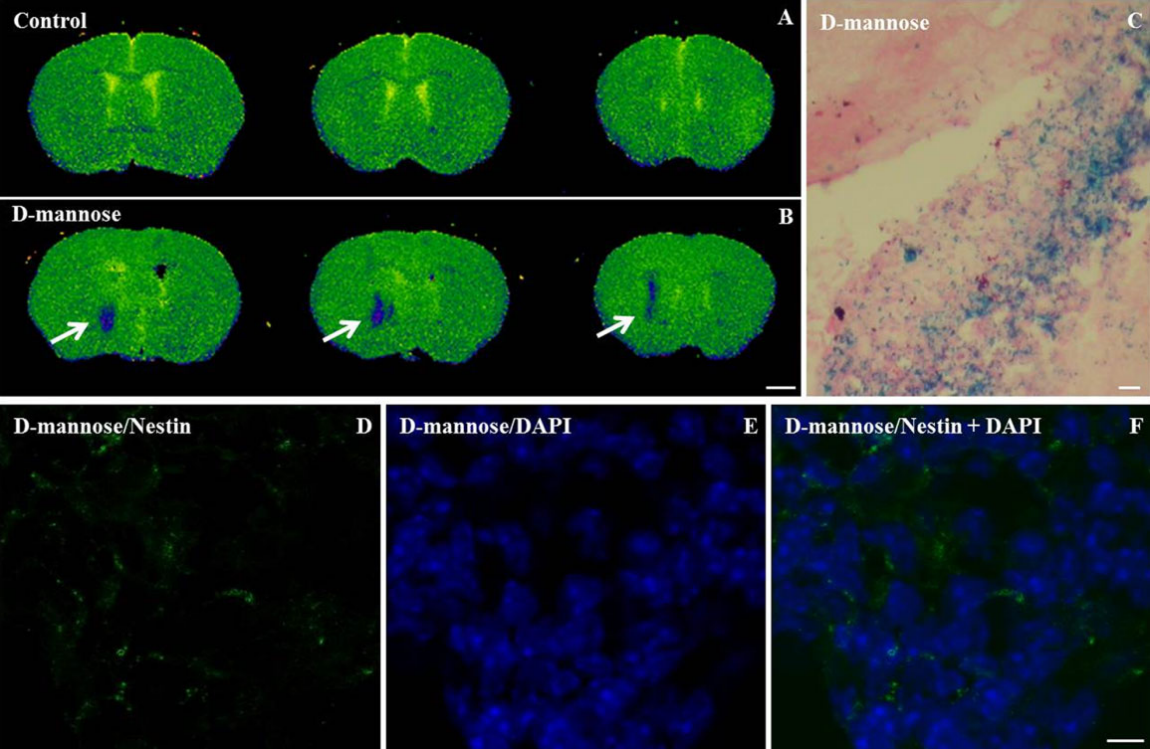
D-mannose(γ-Fe_2_O_3_)-labeled neural stem cells (NSCs) were efficiently detected by *ex vivo* magnetic resonance imaging (MRI), Prussian blue staining and immunohistochemistry. Calculated T2 maps MR images of isolated adult mice brains at 72 h after a unilateral striatal transplantation of (A) unlabeled control neural stem cells and (B) D-mannose(γ-Fe_2_O_3_)-labeled (0.02 mg/ml) neural stem cells show the hypointense signal (blue) at the location of the graft (arrow). Prussian blue staining positive for D-mannose(γ-Fe_2_O_3_) nanoparticles (C) performed on serial rostrocaudal sections cut from the same brain scanned with MRI confirms the location of grafted labeled cells. Immunostaining against nestin (neural stem cell marker, green; D), DAPI (nuclear stain, blue; E) and merged (F) performed on same rostrocaudal sections cut from the same brains confirms the location of grafted cells. MRI scale bar 1 mm. IHC and Prussian Blue scale bars: 10 µm.

Having established an optimized labeling with D-mannose(γ-Fe_2_O_3_)-coated nanoparticles (0.02 mg/ml for 48 h) NSCs were transplanted into the mouse striatum. MRI was performed *ex vivo* and unlabeled NSCs were used as a control. A pronounced hypointense region attributable to D-mannose(γ-Fe_2_O_3_)-labeled NSCs was observed in the striatum in T_2_-weighted images (Fig. 5B). The MRI nanoparticle hypointense signal allowed the visualization and localization of transplanted NSCs labeled with D-mannose(γ-Fe_2_O_3_) nanoparticles within the anatomically defined region of the transplanted tissue with a high spatial resolution. No MRI contrast signal was detected when unlabeled cells were transplanted in the control animals.

To verify if the MRI hypointense signal can be attributed to the D-mannose(γ-Fe_2_O_3_)-labeled NSCs, Prussian blue staining, and immunofluorescence were subsequently performed on corresponding serial rostrocaudal sections cut from the same brains. The formed Prussian blue precipitates showed the presence of the iron nanoparticles distributed in the transplant region (Fig. 5C). Three days after transplantation, NSCs were still nestin positive, reflecting their immature phenotype (Fig. 5F).

### D-mannose(γ-Fe_2_O_3_) Biocompatibility is Similar to Uncoated Nanoparticles

**Figure 6.**
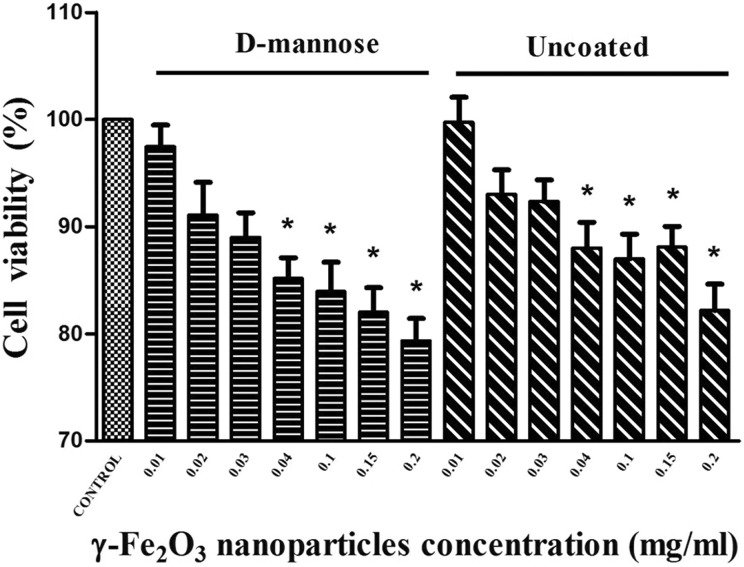
Neural stem cell (NSC) metabolic activity/viability is similarly affected when labeled with D-mannose(γ-Fe_2_O_3_) or Uncoated(γ-Fe_2_O_3_) nanoparticles. NSCs were incubated for 48 h in ascending concentrations of D-mannose(γ-Fe_2_O_3_) or Uncoated(γ-Fe_2_O_3_) nanoparticles (*n*=12). Control NSCs were not treated. Cell metabolic activity/viability was determined by MTT tetrazolium assays immediately after incubation. *p < 0.05, when compared with control.

To compare the effects of D-mannose(γ-Fe_2_O_3_) versus Uncoated(γ-Fe_2_O_3_) nanoparticles on NSCs, the MTT tetrazolium and CalceinAM/PI assays were applied. The MTT assay showed viable cells with active respiratory mitochondrial activity (as mitochondrial succinic dehydrogenases reduce MTT into an insoluble purple formazan)^57^. Both nanoparticle types decreased the number of active/viable NSCs in a dose-dependent manner (Fig. 6). The concentrations higher than 0.03 mg/ml gave significantly different results compared with untreated control cells. The decrease in cell viability was around 20% when the highest concentration of 0.2 mg/ml of nanoparticles was used.

**Figure 7.**
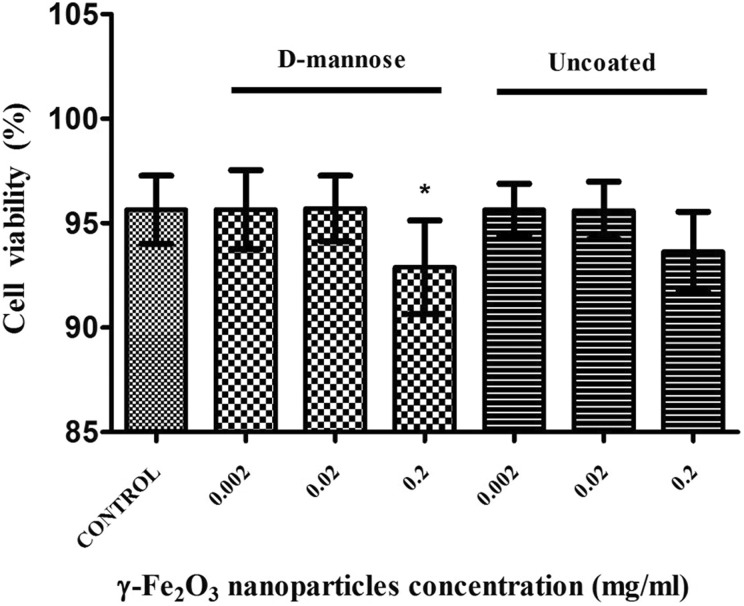
Neural stem cell (NSC) viability is similarly affected when labeled with D-mannose(γ-Fe_2_O_3_) or Uncoated(γ-Fe_2_O_3_) nanoparticles. NSCs were incubated for 48 h in the absence (control) or presence of ascending concentrations of D-mannose(γ-Fe_2_O_3_) or Uncoated(γ-Fe_2_O_3_) nanoparticles. The cell viability was determined by flow cytometry (CalceinAM/PI cytotoxicity assay). *p < 0.05, when compared with control. There were no significant differences between same concentrations of D-mannose(γ-Fe_2_O_3_) and Uncoated(γ-Fe_2_O_3_) nanoparticles.

The CalceinAM/PI assay assessed the percentage of living cells (labeled with Calcein AM) and dead cells (labeled with PI). The mean number of living NSCs treated with D-mannose(γ-Fe_2_O_3_) or Uncoated(γ-Fe_2_O_3_) nanoparticles in all the tested concentrations were higher than 90% (Fig. 7). Both nanoparticles at the highest concentration tested (0.2 mg/ml) showed a significant decrease in NSC viability, but it was only less than 3%. Subsequently, although D-mannose-coated featured similarly in these tests to uncoated nanoparticles, due to the better labeling features, they were chosen for further biocompatibility testing.

**Figure 8.**
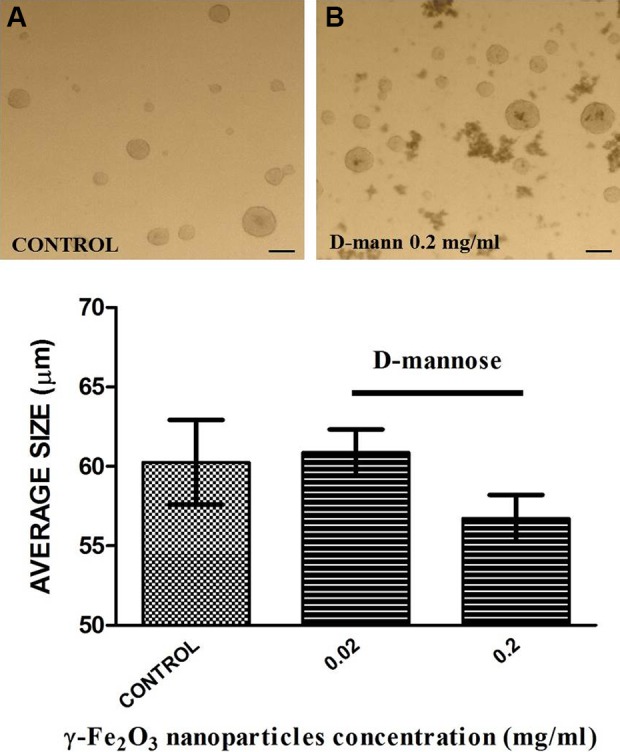
D-mannose(γ-Fe_2_O_3_) labeling does not affect the neural stem cells (NSCs) potential to form neurospheres. Representative image of neurospheres formed from mouse NSC monolayers treated with D-mannose(γ-Fe_2_O_3_) nanoparticles for 48 h (B) or untreated controls (A). Bar chart showing the average sphere diameter per well. Scale bar 50 µm.

Neurosphere morphology was tested to verify if D-mannose coating influenced the NSC differentiation. The D-mannose(γ-Fe_2_O_3_) labeling slightly affected NSC potential to form neurospheres (Fig. 8). All spheres showed round or oval morphology, but their diameters were lower in D-mannose(γ-Fe_2_O_3_) treated culture when 0.2 mg/ml concentration was used (61.33± 1.08 μm vs. 57.12± 1.37 μm, *p* < 0.01).

**Figure 9.**
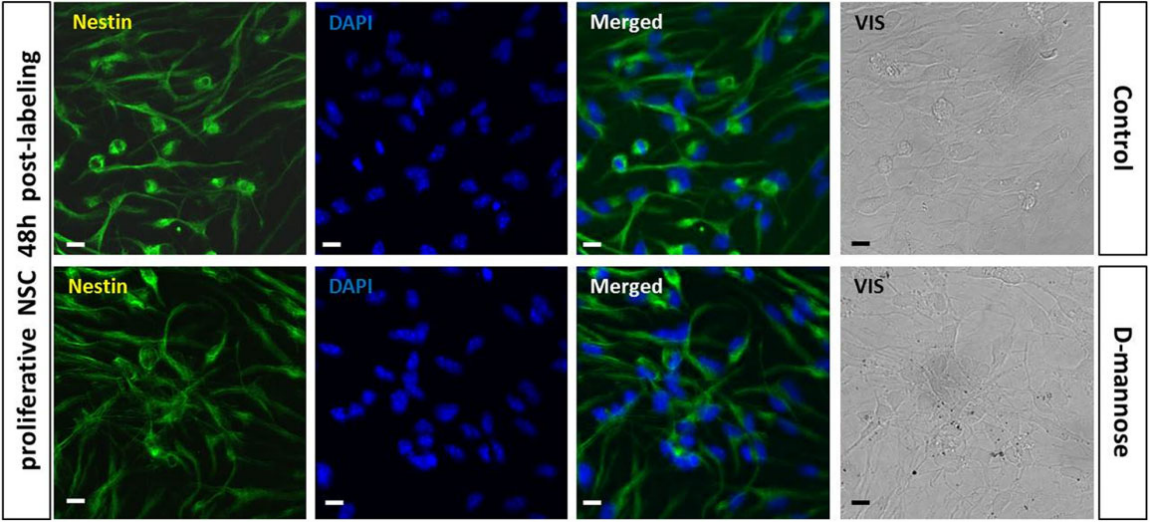
Labeling with D-mannose(γ-Fe_2_O_3_) nanoparticles does not alter neural stem cell (NSC) stemness. The neural progeny of NSCs labeled with 0.02 mg/ml D-mannose(γ-Fe_2_O_3_) nanoparticles was confirmed by immunostaining against nestin (green, NSC marker). Control NSCs were untreated. DAPI was used as a nuclear stain (blue). Scale bars: 10 µm.

**Figure 10.**
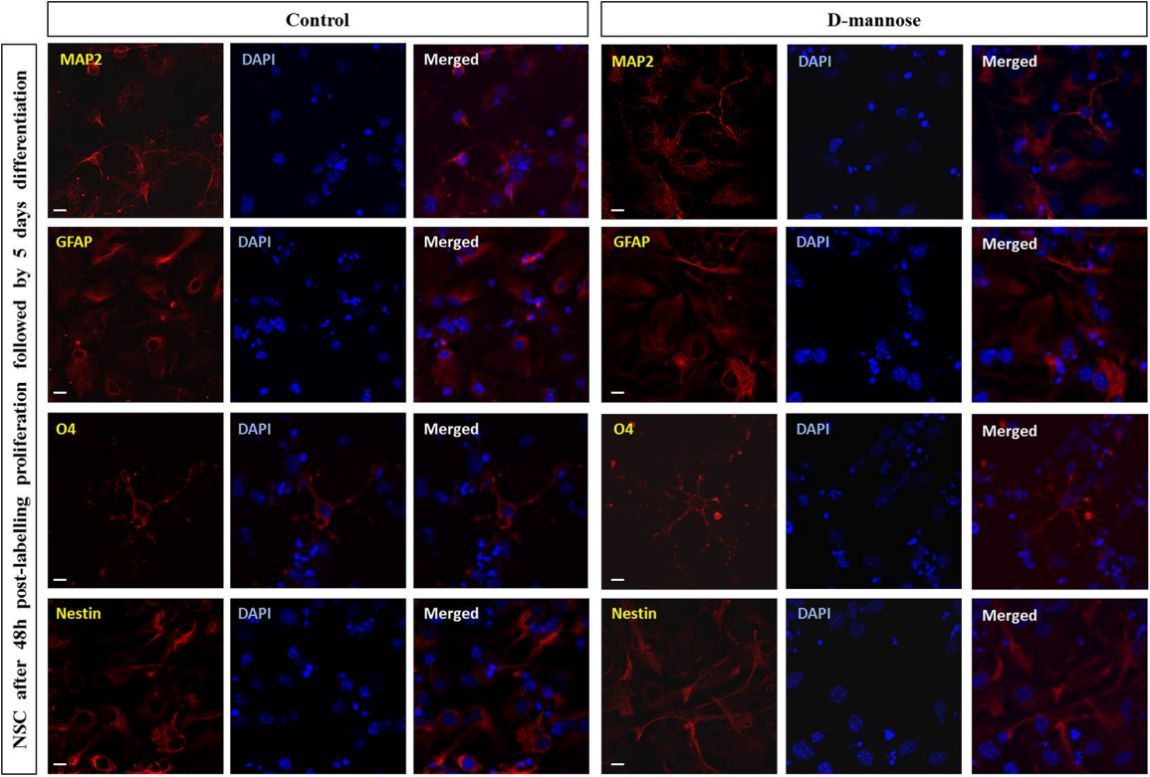
Labeling with D-mannose(γ-Fe_2_O_3_) nanoparticles did not affect the multipotency of neural stem cells (NSCs). The presence of neurons (MAP2+), astrocytes (GFAP+), and oligodendroglial cells (O4+) in the differentiated cultures was assessed by immunofluorescence assay. Significant differences were not observed in the relative proportions of the different neural cell types or in their morphology when NSCs were treated with 0.02 mg/ml of D-mannose(γ-Fe_2_O_3_) nanoparticles with respect to untreated controls. Scale bars: 10 µm.

The differentiation potential of the NSCs after 0.2 mg/ml D-mannose(γ-Fe_2_O_3_) treatment was further analyzed by immunocytochemistry of the resulting cell lineages. The D-mannose(γ-Fe_2_O_3_)-labeled NSCs stained positive for nestin, showing no change in their neural progenitor fate in comparison to control unlabeled cells (Fig. 9). After culturing these cells for further 5 days they readily differentiated to astrocytes (GFAP+), oligodendrocytes (O4+), and neurons (MAP2+) in a similar way as untreated control cells (Fig. 10).

## Discussion

The comparisons of D-mannose(γ-Fe_2_O_3_) to Uncoated(γ-Fe_2_O_3_) nanoparticles performed in this study confirmed that both types of nanoparticles label NSCs, but the labeling is more abundant by the same concentration of D-mannose(γ-Fe_2_O_3_) in the surrounding medium. As D-mannose coating significantly improved nanoparticle internalization compared with the uncoated nanoparticles, it could be considered as a suitable candidate for MRI detection after cell transplantation.

The mechanism of internalization was similar for both types of nanoparticles, as the labeling was affected only by cytochalasin D, an inhibitor of actin-dependent macropinocytosis. There was no effect of other inhibitors, phenylarsine oxide (the clathrin-mediated endocytotic pathway), nocodazole (microtubule-disrupting agent), or filipin (caveolae-mediated endocytotic mechanism)^53^. Still, as shown by TEM, the cellular location or nanoparticles after labeling was not the same, as D-mannose(γ-Fe_2_O_3_) nanoparticles were detected within the vesicles, but Uncoated(γ-Fe_2_O_3_) nanoparticles were dispersed in the cell cytosol. Endocytosis as an internalization mechanism could be divided into two major mechanisms: phagocytosis of foreign materials larger than 750 nm and pinocytosis for nanoparticles or solubles, which can be further divided into clathrin- or caveola-dependent endocytotic mechanisms and macropinocytosis^53^. TEM analysis did not show D-mannose(γ-Fe_2_O_3_) nanoparticles in small vesicles, which would indicate the involvement of clathrin-mediated endocytosis or caveolin-mediated endocytosis types. The observed vesicle diameter of over 500 nm suggested macropinocytosis as the main internalization mechanism^58,59^, the same as shown by the cytochalasin D inhibition, as an inhibitor of actin-dependent macropinocytosis. Uncoated(γ-Fe_2_O_3_) nanoparticles, internalization of which was inhibited in a similar way by cytochalasin D, were not afterward observed in vesicles but scattered in the cytosol. It could be that the positive vesicles were just not identified in the given samples, but we could also speculate that Uncoated(γ-Fe_2_O_3_) nanoparticles were released from the vesicles by previously suggested lysosomal metabolism^53,60–63^.

The vesicle release of Uncoated(γ-Fe_2_O_3_) nanoparticles could indicate eventual higher toxicity, but in this study we have shown that the effects on the cells were comparable for both types of nanoparticles. It should be noted that the effects were comparable for the same concentration of nanoparticles added to the cell medium. The labeling concentration used was consistent with previous studies that found that SPION efficiently labels stem cells without inducing cytotoxicity up to a concentration of 0.2 mg/ml^49,52,64,65^. The labeling and uptake of the D-mannose(γ-Fe_2_O_3_) nanoparticles was higher than of Uncoated(γ-Fe_2_O_3_) nanoparticles, but reaching only up to 50% cell labeling. Previous reports claim reaching up to 95% labeled stem cells with commercially available Molday ION Rhodamine-B™ (MIRB), but no quantitative proof was provided for NSC labeling since only Prussian Blue staining was performed^66,67^. When used for NSC labeling, MIRB showed reduction of the survival, proliferation, and differentiation rate of NSCs with immune response upregulation, which was not the case when used as a mesenchymal stem cell label^66–68^. D-mannose nanoparticles are composed of an iron oxide core coated with D-mannose to prevent nanoparticle aggregation and precipitation. Once ingested by macrophages, the iron oxide core could be metabolized and reused for hemoglobin synthesis. On the other hand, the D-mannose shell could bind to the macrophage mannose receptors (MNR). MNR as a type I transmembrane C-type lectin appeared as an important component of the innate immune system, participating in host defense following infections, specifically through activation of macrophages^69^. MNR is also involved in the innate immune response of healthy and injured nerve tissue, as it was found to be present in microglia, astrocytes, immature neurons, Schwann cells, and olfactory ensheathing cells^70–73^. MNR is involved in receptor-mediated phagocytosis, recognition and clearance of endogenous ligands, cell adhesion, stimulation of cytokine secretion, and antigen transport^74^. However, since the mechanisms of the different brain cell-specific MNR functions still have to be elucidated, we can only speculate on the immunological outcome of the mannosylated nanoparticle MNR activation. In addition to the side effects of applied nanoparticles showed in this study, the subtle changes after cell treatment with maghemite nanoparticles including D-mannose coated were already noticed in the previous studies. The oxidant/antioxidant status of NSCs labeled with the different SPIONs was assessed by measuring GSH and SOD levels, GPx activity, mitochondrial and cell membrane fluidity and permeability, and analysis of DNA damage. The surface coating does not prevent the toxic effects of SPIONs, and different SPION types affect the NSCs similarly^48,49,75–78^. Both *in vitro* immunocytochemical and neurosphere assay analysis of D-mannose labeled NSCs did not show alterations of the neural stem cell identity or changes in NSC multipotency. However, long-term *in vivo* studies should be performed to address their progeny and regenerative capacity after grafting. In agreement with our work, different studies have examined NSC biology after iron oxide agents Ferridex or Endorem labeling, showing no significant differences between the viability, fate, and migratory capacity of labeled and unlabeled NSCs^9,79^. On the other hand, in contrast to our current findings, long-term assessment of MIRB-labeled NSCs showed significantly reduced proliferation and differentiation capacity^66^. Due to the concerns arising from possible toxicity of the nanoparticles, it would be opportune to transplant grafts containing a smaller fraction of trackable labeled cells, allowing the unlabeled cells to perform their therapeutic actions. The transplanted cells labeled by D-mannose(γ-Fe_2_O_3_) nanoparticles were suitable for MRI identification. Although the implantation coordinates were chosen in accordance to previous studies, recent studies show that the transplantation site is crucial for the graft survival, suggesting implantation into the cortex could be even better than in the striatum due to prolonged graft survival^80–83^. MRI was able to assess the precise position of the grafted cells 72 h after transplantation. Previous studies have shown that magnetically labeled cells maintained their contrast up to 3 months after transplantation^84^.

This study has several limitations. First, since the major aims of our study were to assess the feasibility of labeling NSCs with D-mannose nanoparticles, their biocompatibility and their detection by MRI, cell fate was only evaluated at one time point. Prussian Blue and Nissl staining confirmed the localization of the MRI signal. However, this dual staining does not discern between grafted cells and possible intrinsic stem cells, which could have migrated to the lesioned area. As a result, potential benefits or pitfalls of the NSC D-mannose-labeled grafting were not extensively investigated. Second, although multiple cell types would enhance the predictive power of nanosafety assessment, for the abovementioned reason only one cell type was investigated^85^. We confirmed the feasibility of the envisaged labeling strategy, but further studies are needed to evaluate the long-term *in vivo* efficacy of D-mannose NSC labeling, their survival, immunophenotype, and therapeutic potential. In conclusion, D-mannose(γ-Fe_2_O_3_) nanoparticles labeled NSCs more efficiently than uncoated nanoparticles, and were confirmed as an appropriate MRI contrast agent for cell-tracking experiments. The D-mannose(γ-Fe_2_O_3_) nanoparticles labeled NSCs through macropinocytosis did not influence the *in vitro* neural stem cell identity, progenitor activity, and multipotency. However, the subtle changes in cell proliferation and viability were noticed, and were comparable to those induced by uncoated nanoparticles.
